# Accumulation and dissemination of prion protein in experimental sheep scrapie in the natural host

**DOI:** 10.1186/1746-6148-5-9

**Published:** 2009-02-25

**Authors:** Stephen J Ryder, Glenda E Dexter, Lindsay Heasman, Richard Warner, S Jo Moore

**Affiliations:** 1 Department of Pathology, Veterinary Laboratories Agency, Woodham Lane, New Haw, Addlestone, Surrey, KT15 3NB, UK; 2 ADAS UK Ltd, High Mowthorpe, Duggleby, North Yorkshire, YO17 8BP, UK

## Abstract

**Background:**

In order to study the sites of uptake and mechanisms of dissemination of scrapie prions in the natural host under controlled conditions, lambs aged 14 days and homozygous for the VRQ allele of the PrP gene were infected by the oral route. Infection occurred in all lambs with a remarkably short and highly consistent incubation period of approximately 6 months. Challenge of lambs at approximately eight months of age resulted in disease in all animals, but with more variable incubation periods averaging significantly longer than those challenged at 14 days.

This model provides an excellent system in which to study the disease in the natural host by virtue of the relatively short incubation period and close resemblance to natural infection.

**Results:**

Multiple sites of prion uptake were identified, of which the most important was the Peyer's patch of the distal ileum.

Neuroinvasion was detected initially in the enteric nervous system prior to infection of the central nervous system. At end stage disease prion accumulation was widespread throughout the entire neuraxis, but vacuolar pathology was absent in most animals that developed disease at 6–7 months of age.

**Conclusion:**

Initial spread of detectable PrP was consistent with drainage in afferent lymph to dependent lymph nodes. Subsequent accumulation of prions in lymphoid tissue not associated with the gut is consistent with haematogenous spread. In addition to macrophages and follicular dendritic cells, prion containing cells consistent with afferent lymph dendritic cells were identified and are suggested as a likely vehicle for carriage of prions from initial site of uptake to the lymphoreticular system, and as potential carriers of prion protein in blood. It is apparent that spongiform change, the characteristic lesion of scrapie and other prion diseases, is not responsible for the clinical signs in sheep, but may develop in an age dependent manner.

## Background

Scrapie is a transmissible spongiform encephalopathy (TSE), an infectious neurodegenerative disease of sheep, goats and moufflon, endemic in many countries of the Northern hemisphere and characterised by extremely long incubation periods, frequently of many years. As a disease of sheep, scrapie has been known for centuries in Europe. The emergence of bovine spongiform encephalopathy (BSE) in the UK [1], a disease of cattle possibly derived from scrapie that has given rise to a new form of Creutzfeldt-Jakob disease in humans, vCJD [2], has raised the profile of scrapie in sheep.

Etiologically scrapie has been variously thought of as hereditary or due to a slow virus infection. The cause is now thought to be a prion, an abnormal isoform of the host's own cellular prion protein PrP^C^, misfolded to give PrP^Sc^, probably the sole constituent of the infectious prion [3]. Infection with scrapie prions follows a familial pattern and is known to be associated with susceptible alleles of the prion protein gene [4].

Infection with the agent is widespread throughout lymphoid and central nervous system tissues of the body of infected sheep, but the clinical signs are of a nervous disease and typically feature changes in behaviour, pruritus, ataxia, trembling and weigh loss leading ultimately and invariably to death.

Studies on the pathogenesis of scrapie as a natural infection in sheep have shown that the infectious agent accumulates in tissues of the gut and lymphoreticular system early in the incubation period, preceding invasion of the nervous system [5]. More recently, and following the development of the prion hypothesis to account for the infectious agent, more detailed studies which use detection of the prion protein have been possible, in preference to bioassays. Such studies, using immunohistochemistry applied to sheep naturally infected with scrapie, have enabled the distribution of prion protein throughout the incubation period to be mapped at the cellular level [6,7], and have considerably enhanced current understanding of the natural disease. In all such studies however, the timing of infection is unknown as routes and mechanisms of transmission of natural scrapie are obscure.

It is generally thought that sheep become infected early in life, but in natural disease the exact timing of infection is never known and infection may occur repeatedly throughout the course of the disease; adult sheep are certainly susceptible to scrapie infection [8]. The route of infection is likely to be oral in the majority of cases of natural scrapie, based on either neural targeting of the agent [9] or initial detection of infection in lymphoid tissues of the gastrointestinal tract, in particular the Peyer's patches (PP) of the distal ileum [6,7]. Initial uptake from the gut lumen probably occurs by transcytosis of prions by specialised epithelial cells, M cells. Replication and accumulation of the agent is associated with macrophages and follicular dendritic cells in advance of detectable PrP in the enteric nervous system, which in turn precedes invasion of the central nervous system, the onset of clinical disease and the vacuolar pathology typical of scrapie and related disorders. Exact mechanisms of spread to lymphoid tissues are uncertain. Infection of blood has long been suspected and has recently been demonstrated unequivocally in experimental BSE infection of sheep[10].

Due to the difficulties of working with the natural disease, in particular the extremely long incubation periods, rodent models of scrapie infection have been widely used to study pathogenesis, and show many similarities to the natural disease. Whilst the use of rodents has many advantages and has been highly productive, it is apparent that most natural prion infections occur in ruminants. There are clear limitations to studying the pathogenesis in monogastric rodents that do not appear to be able to transmit the disease naturally, although precise knowledge of the timing of infection in experimental models has advantages.

Experimental infections of sheep with scrapie have used predominantly parenteral challenges; the current understanding of genetic susceptibility of sheep to scrapie was established using susceptibility to subcutaneous inoculation [11,12]. Challenges using the presumed natural (oral) route of challenge have been reported in the literature. One study used 5 month old sheep homozygous for the ARQ allele of the PrP gene, not all of which succumbed to disease following challenge with an inoculum made from spleen and brain of affected sheep [12,13]. Oral challenge of lambs homozygous or heterozygous for the VRQ allele of the PrP gene showed accumulation of prion protein in the PP when maintained for up to 11 months post challenge, but in that study none were allowed to progress to clinical disease [14]. Oral challenge of ARQ homozygous sheep with the BSE agent has been used to study pathogenesis of BSE infection of sheep [15,16]. In this study a sequential accumulation of disease specific PrP was detected in lymphoid tissues prior to neuroinvasion, very similar to that occurring in scrapie. No such detailed study of scrapie pathogenesis in the natural host under carefully controlled conditions has been described.

We report an experimental model of scrapie infection in which scrapie-free sheep of known genetic susceptibility were challenged orally at a young age, before weaning. In this model there was a rapid onset of disease, leading to clinical scrapie after a precisely known and remarkably short incubation period, averaging 188 days (time to euthanasia). The model provides the known time of infection and high consistency found in rodent models, but in the natural host and with comparable or shorter incubation periods. The experimental disease closely resembled the natural disease, confirming this as an ideal model for studying scrapie under controlled conditions. Immunohistochemistry for disease specific PrP (PrP^d^) enabled the distribution and spread of the infection to be described in detail throughout the incubation period.

## Results

### Clinical disease

**Figure 1:**
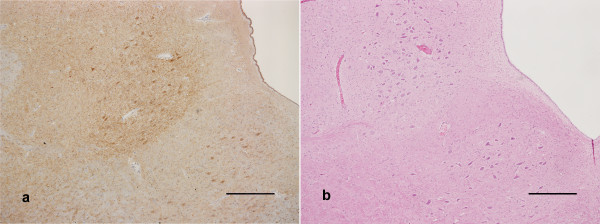
**Medulla oblongata at the level of the obex**. a) Widespread immunolabeling of moderate to marked intensity in the hypoglossal nucleus (bottom right) and dorsal motor nucleus of the vagus nerve (centre). Scale bar 500 μm. b) Equivocal vacuolation in the ventral border of the dorsal motor nucleus of the vagus nerve. Scale bar 500 μm.

Initial onset of clinical signs, in particular changes in behaviour and a subtle increase in nervousness, was noted from approximately 4 months post infection (mpi). Increased nervousness, pruritus and ataxia were commonly observed, and occurred in all scrapie-challenged sheep except those killed at pre-determined time points early in the incubation period. Table 1 shows incubation periods for each group. Euthanasia as a result of onset of definite clinical signs was carried out after a mean interval of 185 days in the 5 g dose group and 192 days in the 1 g dose group. Disease was confirmed by identification of widespread PrP^d ^accumulation in the CNS by immunohistochemistry (Fig 1a) and detection of proteinase K resistant PrP^Sc ^by Western blotting.

**Table 1:** Incubation periods

Group	Challenge	Age at challenge (days)	Attack rate	Mean incubation period (days) Range, (SD)
1	1 g RBP1 oral	14	25/25	192 185–204, (6.9)
2	5 g RBP1 oral	14	28/28	185 178–198, (7.4)
3	1 g RBP1 oral (after 3 g scrapie negative sheep brain)	245	8/8	345 198–639, (186)
4	Exposure to groups 1 and 2	42 days onwards	4/4	649* 592–716, (61)
5	3 g Scrapie negative sheep brain	14 days	8/8	1085** 880–1231 (156)

* Days since mixing with groups 1 and 2

** Age at death, as exact timing of infection unknown

### Cellular distribution of PrP^d ^in lymphoid tissues

**Figure 2:**
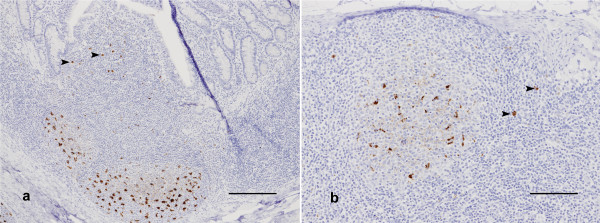
**Immunolabeling in lymphoreticular tissues**. a) Distal ileum. Large, intensely labelled PrPSc deposits in macrophages in the germinal centres of Peyer's patch follicles (centre bottom) and the overlying dome (arrows). Diffuse, branching PrP^d ^deposits are consistent with labeling of follicular dendritic cells. Scale bar 100 μm. b) Mesenteric lymph node. Macrophage and follicular dendritic cell labeling, as well as putative veiled afferent dendritic cells in the subcapsular and interfollicular areas (arrows). Scale bar 100 μm.

Three types of cell labelled for PrP^d ^could be distinguished in lymphoid tissues, based on morphology, PrP immunolabeling and location:

The predominant cell type labelled was a large cell with irregular shaped nucleus, containing one or several large and intensely labelled deposits of PrP (Fig 2). Based on these features these cells are tentatively identified as macrophages, and were detected within germinal centres of all lymphoid tissues, the inter-follicular areas of tonsil and lymph nodes, germinal centres and marginal zones of the spleen and subcapsular sinuses of lymph nodes.

The morphology and location of some immunolabelled cells in the domes of Peyer's patches (Fig 2a), subcapsular sinus of lymph nodes, and some interfollicular areas (Fig 2b) are suggestive of dendritic cells.

Branching patterns of immunolabeling of moderate intensity and even distribution but difficult to associate with individual nuclei were commonly detected in germinal centres (Fig 2). This was tentatively identified as immunolabeling of follicular dendritic cells (FDC).

### Anatomical distribution of PrP^d ^in gut associated lymphoid tissues

Table 2 shows the timing of detection of PrP^d ^in tonsils, PP and draining lymph nodes. Due to the similarity of the incubation periods and disease progression in the 5 g and 1 g dose groups, data from both are considered together in the results. No PrP^d ^was detected in animals killed at 3, 8 and 14 days post infection (dpi). Initial detection of PrP^d ^was in the PP of the distal ileum, in a single animal 21 dpi and in 5/6 animals at 28 dpi. In these animals this consisted of immunolabeling of a very small number of lymphoid follicles only. Immunolabeled macrophages were observed in the follicles of the PP, and less commonly in the domes. Immunolabeling was confined to a small number of cells within each PP follicle and only a minority of follicles showed any positive cells.

**Table 2:** Disease specific PrP (PrP^d^) detection in gastrointestinal tract and associated lymphoid and nervous tissues in pre-clinical stages of disease

Tissue	Time post infection (days) Number positive/number examined					
	(21)	(28)	(55)	(81)	(110)	(147)
						
Distal ileum Peyer's patch	1/3	5/6	8/8	10/10	10/10	3/3
Jejunum Peyer's patch	0/3	1/4	6/8	8/10	9/9	3/3
Mesenteric lymph node	0/3	0/6	1/8	7/10	10/10	3/3
Palatine tonsil	0/3	1/6	4/7	9/10	9/10	3/3
Pharyngeal tonsil	0/3	0/6	3/8	8/10	10/10	2/3
Medial retropharyngeal lymph node	0/3	1/6	5/8	10/10	10/10	3/3
Lateral retropharyngeal lymph node	0/3	Nd	0/5	3/10	7/9	3/3
Enteric nervous system	0/3	0/6	0/8	0/10	10/10	2/3

nd: not done

No disease specific PrPd detected in any animals killed at 3, 8 and 14 days post infection.

In addition to the distal ileum, PrP^d ^was also detected in PP of jejunum in one animal at 28 dpi.

In the palatine tonsil of one lamb at 28 dpi just two lymphoid follicles and numerous cells scattered between the positive follicles and the tonsil sinus were labelled.

In another lamb there was immunolabeling of the medial retropharyngeal lymph nodes, involving only a few positive cells within a few follicles. In this lamb there was no immunolabeling in either the palatine or pharyngeal tonsil.

In three control sheep killed at 27 dpi there was no PrP immunolabeling in any tissues.

At 55 dpi PrP^d ^was more widespread, involving a greater number of PP follicles in the distal ileum and to a lesser extent in the jejunum. The draining lymph nodes of the small intestine, the mesenteric lymph nodes, were positive in only one of these sheep. The palatine, and to a lesser extent pharyngeal, tonsils were positive at this time point, as were the draining lymph nodes, the medial retropharyngeal nodes. In palatine tonsils most follicles were negative but the few positive follicles occurred in clusters, not scattered, suggesting a focus of infection entering several follicles, or spread locally to adjacent follicles. In all cases PrP^d ^labeling was detected mainly as large irregular deposits in macrophages within germinal centres of the lymphoid follicles. There was also labeling of some irregularly shaped cells in the extra-follicular areas of tonsils and the cortices of lymph nodes. Occasional immunolabeled macrophages were observed in the subcapsular sinuses of lymph nodes. In all animals at this time point the majority of lymphoid follicles showed no evidence of PrP^d ^accumulation.

At 81 dpi PrP^d ^was detected in all 10 animals killed, and more widely than at earlier time points. Intense labeling for PrP^d ^was present in PP of distal ileum of all sheep, involving several or most lymphoid follicles but some follicles were negative in all sheep. PrP^d ^was also detected in the jejunal PP and in mesenteric lymph nodes of most animals. In PP at this time point PrP^d ^positive macrophages were abundant, and in some follicles there was also prominent labeling of FDC. In some there was also intense immunolabeling of cells in the dome and in the capsule of the follicle. Pharyngeal and palatine tonsils of most sheep were positive and PrP^d ^was detected in the medial retropharyngeal lymph node of all 10, but was rare in the lateral retropharyngeal lymph node, which receives afferent lymph from the medial node. In all of these locations larger numbers of follicles were found to be positive than at earlier time points and the immunolabeling was more intense, involving immunolabeling of both macrophages and FDCs in many follicles. In addition positively labelled cells were observed in the inter-follicular areas and occasionally in the subcapsular sinuses of mesenteric and medial retropharyngeal nodes.

At 110 and 147 dpi PrP^d ^was detectable in most lymphoid tissues associated with the GI tract, and in these tissues most lymphoid follicles were positive. At this time point a combination of macrophages and FDC within follicles showed intense immunolabeling for PrP^d^. Many positive macrophage-like cells were also observed in the inter-follicular areas and subcapsular sinuses of the lymph node cortices, particularly in the medial retropharyngeal and mesenteric nodes.

During the clinical stages of disease intense immunolabeling of both macrophages and FDCs was found in virtually all follicles of all the gut associated lymphoid tissues. Abundant immunolabeled macrophages in the subcapsular sinuses were also observed in many lymph nodes at this time point. These were particularly prominent in the medial retropharyngeal lymph nodes and mesenteric lymph nodes, both of which directly drain heavily affected tissues: the tonsils and small intestine respectively. The lateral retropharyngeal lymph nodes, which receive afferent lymph from the medial retropharyngeal nodes, showed consistently less immunolabeling for PrP^d^.

### Immunohistochemistry of other lymphoid tissues

Timing of detection of PrP^d ^in spleen and in lymph nodes not draining the GI tract is shown in Table 3. No PrP^d ^was detected in animals killed prior to 81 dpi. Initial detection in these tissues was in some sheep only, at 81 dpi. In all cases this involved only occasional lymph nodes, very few follicles in each lymph node and predominantly in macrophages only. At this time point immunolabeling in the spleen was confined to the germinal centres of peri-arteriolar lymphoid sheaths.

**Table 3:** Disease specific PrP (PrP^d^) detection in non-gastrointestinal associated lymphoid tissues

Tissue	Time post infection (days) Number positive/number examined					
	(28)	(55)	(81)	(110)	(147)	(Clinical)
						
Spleen	0/6	0/8	3/10	10/10	3/3	15/15
Broncho-mediastinal lymph node	nd	0/8	3/10	10/10	3/3	9/10
Tracheo-bronchial lymph node	nd	0/8	2/10	8/9	3/3	8/8
Popliteal lymph node	0/6	0/8	0/10	9/10	3/3	10/10
Pre-scapular lymph node	nd	0/8	2/10	7/8	3/3	10/10
Inguinal lymph node	nd	0/8	4/10	10/10	3/3	9/10
Submandibular lymph node	nd	0/8	2/10	10/10	3/3	10/10

nd: not done

At 110 dpi, 147 dpi and in clinical disease PrP^d ^was widespread and was detected in most lymphoid tissues. The intensity and extent of PrP^d ^detection varied but at least some follicles were labelled in all lymph nodes examined from all animals.

In the spleen the extent and intensity of immunolabeling of PrP was less than in other lymphoid tissues. Small numbers of macrophages were labelled within germinal centres and marginal zones of many, but not all, periarteriolar lymphoid sheaths.

At clinical disease much more intense labeling was detected, in some germinal centres in the spleen including FDCs and macrophages, similar to that seen in lymph nodes. Many lymph nodes including popliteal and pre-scapular lymph nodes, which receive afferent lymph from the hind and forelimbs respectively, also showed labelled macrophages in the subcapsular sinuses.

### PrP^d ^accumulation in inflammatory lymphoid tissue

**Figure 3:**
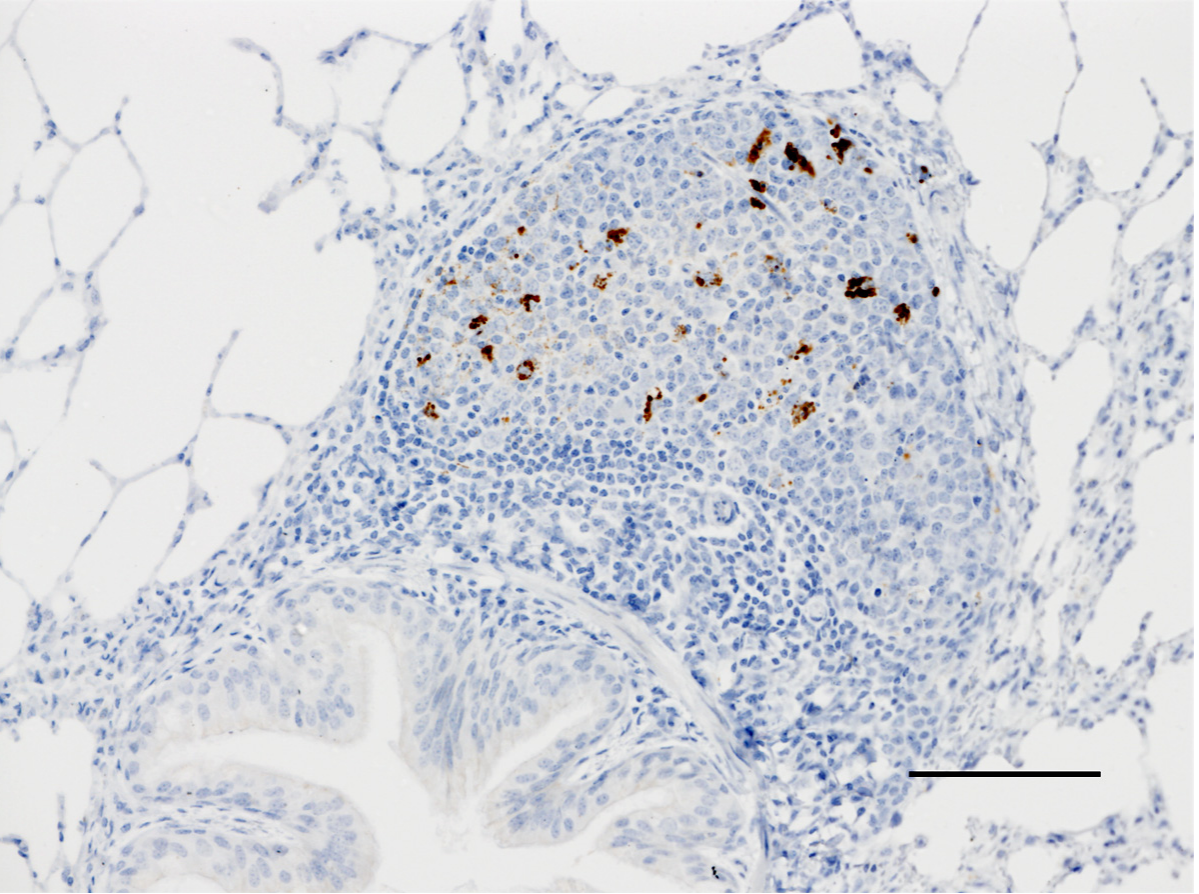
**Immunolabeling of macrophages in a lymphoid follicle in the lung**. Scale bar 100 μm.

In some sheep, in which transient episodes of incidental pneumonia had occurred, there was hypertrophy of the bronchial associated lymphoid tissue giving rise to germinal centre formation. In all such cases in scrapie challenged sheep there was accumulation of PrP^d ^in this organised lymphoid tissue (Fig 3), predominantly in macrophages, similar to that in lymph nodes.

In one sheep there was unilateral enlargement of the submandibular lymph node, probably associated with an (undetected) unilateral inflammatory process in the oral cavity. The enlarged lymph node contained a large number of secondary follicles, in contrast to very few in the much smaller, non-reactive contra-lateral node. In this sheep there was extensive accumulation of PrP^d ^in the reactive node, where all the secondary follicles contained labelled macrophages and FDC, whereas there was virtually no PrP^d ^detectable in the non-reactive lymph node.

### Central nervous system

All animals were similar and showed a PrP^d ^accumulation consisting of a combination of particulate, intra-neuronal, and intra-glial labeling in the grey matter of the spinal cord and brain.

Examination of spinal cord sections at the following levels: cervical segment 8, thoracic segments 1, 6 and 12 and lumbar segment 4, showed no infection at 110 dpi, but the brain stem was infected at this time point in 7/10 sheep. In all cases this was confined to weak immunolabeling of neurons along the ventral border of the dorsal motor (parasympathetic) nucleus of the vagus nerve, and was bilaterally symmetrical. At 147 dpi there was a greater involvement of the brain stem; all neurons of the dorsal motor (parasympathetic) nucleus of the vagus nerve showed fine particulate deposits and there was widespread but weak immunolabeling of glia and some neurons throughout the medulla (Fig 1a). Small amounts of labeling were seen in the spinal cord, intra-glial labeling at most levels in the intermedio-medial grey matter, and neuronal labeling of the intermedio-lateral grey matter, the source of sympathetic outflow, in all three animals examined.

At end stage disease the extent of PrP^d ^distribution in the brain had increased to involve all grey matter areas. Weak particulate labeling of the perikaryon was observed in virtually all neurons, together with intra-glial deposits. No large accumulations of PrP^d ^amyloid plaques were observed and neuropil labeling was mild (Fig 1a). The spinal cord showed a similar pattern of perikaryonal and intra-glial deposits throughout the grey matter at end stage disease.

Histopathological examination of brains showed minimal or no lesions in the medulla oblongata at the level of the obex (Fig 1b), the site most typically affected by vacuolar pathology in scrapie affected sheep [18]. Elsewhere in the brain vacuolar pathology was sparse or absent, but in some sheep vacuolation of the hypothalamus was identified, even in the absence of vacuolation in the medulla oblongata.

### Peripheral nervous system

Table 4 shows the timing of detection of PrP^d ^in peripheral nervous tissues. No labeling of PrP in the enteric nervous system was seen prior to 110 dpi. Initial involvement of the peripheral nervous system was detected in the myenteric and submucosal plexi of the gastro-intestinal tract. At 110 dpi this was present in distal ileum in all cases, and in the jejunum, colon and caecum of some cases. At 147 days the pattern was similar, but one animal did not show PrP^d ^in the distal ileum. At end stage disease the extent of involvement had spread to include plexi in the jejunum and colon of all cases, and of the caecum and duodenum of most cases, as well as the abomasum of five and the rumen of one case.

**Table 4:** Detection of PrP^d ^in peripheral tissues from 110 days post-inoculation

Enteric nervous system of:	Time post infection (days) Number positive/number examined		
	(110)	(147)	(Clinical stage)
			
Distal ileum	10/10	2/3	10/10
Jejunum	3/10	1/3	10/10
Colon	7/10	2/3	9/9
Caecum	1/10	1/3	9/10
Duodenum	0/10	0/3	9/10
Oesophagus	0/10	0/3	0/10
Rumen	0/10	0/3	1/10
Abomasum	0/10	0/3	5/10
			
Celiaco-mesenteric ganglion	0/3	0/3	3/4
Stellate ganglion	0/7	0/3	2/8
Cranial Cervical ganglion	0/10	0/2	1/7
Sympathetic chain	0/4	0/3	0/7
Nodose ganglion	5/9	1/2	6/6

The only peripheral ganglion in which PrP^d ^was detected at 110 or 147 days was the nodose ganglion, a sensory ganglion embedded within the vagus nerve that contains cell bodies of sensory neurons passing from the gut to the nucleus of the solitary tract in the brain stem. At end stage disease there was more extensive involvement of peripheral ganglia, including sympathetic ganglia in some but not all animals, but in no cases were ganglia of the sympathetic chain involved.

### Unchallenged in-contact lambs

Four unchallenged lambs were mixed in the same pens as dosed animals from 28 days following challenge. The regular cleaning of pens will have avoided any gross contamination of the environment, however in one pen a single lamb was orally dosed after addition of the in-contact lambs. All four succumbed to scrapie at an average of 666 days (638 days post initial exposure), similar to the incubation period in naturally infected sheep of this genotype kept in the flock from which inoculum RBP1 was made. Whether this infection resulted from ingestion of remaining passively excreted inoculum or infection actively shed into the environment, i.e. natural lateral transmission, cannot be determined with certainty.

### Control sheep

Control sheep challenged with 3 g of normal brain homogenate were killed at 13 days (n = 2), 27 days (n = 3), 95 days (n = 5) and 198 days (n = 3) after challenge. The same tissues were examined from control as from challenged sheep. In a single control sheep killed 198 days after challenge a small amount of PrP^d ^accumulation in the mesenteric lymph nodes was detected. No PrP^d ^was detected elsewhere in this animal. This minimal accumulation was consistent with the very early stage disease. All other control sheep were negative on immunohistopathology.

Following the surprisingly short incubation period (4 months) found in lambs challenged at 14 days of age, eight control sheep, which had been previously orally dosed with normal brain, were redosed at approximately 6 months of age with 1 g of RBP1 to investigate whether the short incubation period was related to young age at dosing. All eight sheep eventually developed clinical disease, which was confirmed by the presence of both PrP^d ^and vacuolar pathology in the medulla oblongata. Incubation periods ranged from 198 to 639 days, average 345 days, significantly longer than for challenge at 14 days of age, with a much greater range (Table 1).

The remaining eight control sheep were retained after all challenged sheep had succumbed. All of these sheep developed scrapie and were euthanased at between 880 and 1231 days of age, despite having been challenged with uninfected brain homogenate only and having been kept isolated from challenged sheep as described.

## Discussion and conclusion

The experimental disease produced in this study in VRQ homozygous sheep challenged orally at 14 days of age provided a highly consistent and relatively short incubation period model for the study of scrapie in the natural host. The features of the disease resembled natural scrapie, including spread of infection throughout the lymphoid tissues in the pre-clinical stages of disease, clinical signs and possibly natural transmission.

All of the challenged sheep allowed to survive beyond 16 weeks developed clinical signs at approximately 4 mpi. All progressed to definite clinical signs necessitating euthanasia, with incubation periods between 6 and 7 months after challenge. This is a remarkably short incubation period for scrapie. Even in high incidence flocks age at death is commonly 20–24 months [7,19], and in the flock of sheep from which inoculum RBP1 was made all VRQ homozygous sheep succumb to scrapie at 19–21 months of age [8]. There have been occasional reports of natural cases of scrapie in very young sheep but these are exceptional [20]. The consistently short incubation period in all challenged sheep, whether given 1 g or 5 g of inoculum is similar to the incubation period in subcutaneously challenged sheep [21]. It is suggested that 6–7 months is therefore close to the minimum incubation period for scrapie in sheep. The consistency seen in this study is comparable to that seen in mice challenged with mouse adapted scrapie using overwhelming doses by the intra-cranial route.

Challenge of sheep at 6 months of age resulted in high risk of infection; all sheep developed disease, but with a wide range of incubation periods from 6.5 to 21 months, on average less than that encountered in naturally infected flocks. Therefore it is clear that oral challenge of sheep, regardless of age between 14 days and 6 months, results in a shorter incubation period than natural disease, but challenge earlier leads to greater consistency in the effect of challenge and a shorter average incubation period. This probably reflects higher susceptibility in very young lambs, due to a combination of several factors, including small size giving a high ratio of dose to body weight, a well developed and highly active gut associated lymphoreticular system, and possibly greater patency of the gut to uptake of intact proteins.

Clinically affected sheep all showed typical clinical signs associated with scrapie but those which developed disease at 7 months of age did not have vacuolar pathology in the brain, while those which developed disease at greater ages did. This demonstrates that although vacuolation of the grey matter of the brain and spinal cord is a pathological hallmark of classical scrapie, it is not responsible for clinical signs and does not develop in young animals. This could be due to age related factors which prevent very young animals from developing vacuolar changes, or due to the extremely short incubation period and clinical course in this study. An important implication of this finding is that scrapie should be considered as a differential diagnosis for neurological disease in young animals, and, if typical histological lesions are not present, PrPd detection methods (immunohistochemistry, Western blot etc.) should be applied before ruling out a diagnosis of scrapie.

The distribution of PrP^d ^in the sheep described here is similar to that previously described in natural disease [6,7,22]. Prior to detectable PrP^d ^accumulation in any tissues there was a latent period of between 3 and 4 weeks following challenge, during which no detectable infection, or indeed residual inoculum, was present. In natural disease it is unclear whether this latent period represents the time taken to become infected or a period where infection is present but undetectable. In this model it is clear that animals are infected for 3–4 weeks in the absence of detectable PrP^d ^even using the highly sensitive immunohistochemistry method used here. During this period it is likely that replication takes place but without accumulation of PrP^d ^to levels detectable by current methods. In other genotypes of sheep this latent period would be expected to be longer, perhaps lasting years after natural infection and represents a considerable challenge for the detection of pre-clinically infected sheep in the general or slaughter population.

Following this latent period PrP^d ^was detected in tissues that take up antigen directly from the gastro-intestinal tract; the Peyer's patches and tonsils. The continuous accumulation of PrP^d ^in these tissues, and the clustering of positive follicles demonstrated in particular in the tonsil, indicate local replication and spread between adjacent follicles. Although it is evident that infection occurred at multiple sites, including the palatine tonsil and PP, the PP of the distal ileum were the most frequent tissue to become infected initially. The size of the distal ileal PP in young ruminants is such that it probably represents quantitatively the most important site of uptake.

Initial spread from sites of uptake was to the draining lymph node. The PP drain to the mesenteric lymph nodes and the tonsils to the medial retropharyngeal nodes (MRLN), which in turn drain to the lateral retropharyngeal nodes (LRLN). Labeling in the MRLN was much more intense than in the LRLN, suggesting import of prions in afferent lymph but minimal export of prions from the MRLN in efferent lymph. Cell types labelled in all of these tissues were macrophages and FDCs, as has been reported previously [6]. We postulated that some of the immunolabeled cells in the subcapsular sinuses may have been veiled or afferent lymph dendritic cells (VDC), which are carried in afferent lymph from many tissues to draining lymph nodes and have been shown to carry prions from the gut to draining lymph nodes in an experimental rodent model [23]. However, for technical reasons we have not yet been able to confirm the identity of these cells in the formalin fixed tissues.

PrP^d ^was not observed in any leukocytes in non-lymphoid tissues, blood vessels or lymphatics, but macrophages and putative VDC in secondary lymphoid organs were seen to be infected. As monocytes and macrophages are present in blood and acute inflammation, it would be anticipated that occasional infected cells would be encountered if present, but they were not. Macrophages are clearly involved in local accumulation and transport, and possibly degradation, of prions, but we found no evidence for macrophages acting as vehicles for systemic spread.

Haematogenous spread of the infectious agent is essential to account for the widespread dispersal of the agent to lymphoid tissues that do not drain the gastro-intestinal tract and to the spleen which does not receive afferent lymph. Attempts to detect prions in blood have had variable success [24,25], consistent with infection being present but at low, and possibly varying, levels during the incubation period.

The total quantity of PrP^d ^detected in lymphoid tissue increased as the incubation period progressed. Replication and accumulation can be viewed as occurring in a series of cycles operating in parallel; initially local uptake, replication and accumulation in individual follicles of lymphoid tissue lining the gastrointestinal tract. Subsequently local infection of adjacent lymphoid follicles and spread via lymphatic drainage to dependent lymph nodes occurs; later there is a low level of "leakage" of prion-infected cells from lymph nodes to the circulation, possibly in efferent lymph, leading to haematogenous spread of infection to any secondary lymphoid tissue. Infection of lymph nodes not draining the gut could occur directly from blood and possibly also via extravasation and return to lymphoid tissue in afferent lymph. These cycles continue throughout the incubation period, progressively increasing the amount of prion protein present in all lymphoid tissues, even after neuroinvasion has occurred.

Initial evidence of infection of the nervous system was detection of PrP^d ^in the enteric nervous system of the small intestine, closest to the initial site of infection. As disease progressed, PrP^d ^was found in enteric nervous system distal and proximal to this, suggesting a progressive spread throughout the peripheral nervous system. Transfer of prions to the central nervous system, the crucial precursor to clinical disease, was initially to the brain stem, and specifically to parasympathetic neurons. No direct evidence of transport in peripheral nerves has been found, i.e. PrP^d ^was never detected in nerve fibres, only in cell bodies.

Appearance at specific sites in the CNS implicates parasympathetic fibres in the vagus nerve as transporters, and appearance of PrP^d ^in neurons of the nodose ganglion additionally implicates sensory fibres within the vagus nerve. There was minimal involvement of sympathetic nervous system neurons however until late stage disease, suggesting that central-to-peripheral spread accounts for most infection of sympathetic ganglia. Spread via splanchnic nerves from the gut to the intermediolateral grey columns of the thoracic spinal cord is possible, but due to the late stage at which this occurred, central-to-peripheral spread is difficult to exclude. Therefore the parasympathetic nervous system appears to be the principal route for prions entering the CNS.

The increased amounts of PrP^d ^demonstrated in a reactive compared to non-reactive lymph node, and in chronic inflammatory tissue in the lung, show that the state of activity of the lymphoreticular system has an influence on the pattern of distribution of prion replication/accumulation in lymphoid tissue. Thus any tissue in which chronic inflammation occurs leading to organised lymphoid tissue and germinal centre formation may contain the agent. This observation raises the possibility that intercurrent disease, or even vaccination, which will affect the state of activation of the lymphoid system, might influence the dynamics of prion accumulation; it remains to be determined whether this may lead to any effect on susceptibility to infection or progression of the disease.

The mechanism of natural spread of scrapie is unclear. The only known source of natural shedding of the agent into the environment is the placenta and foetal fluids, in which PrP^Sc ^has been detected on numerous occasions [13,26]. Attempts to identify infectivity in various excretions and secretions have been unsuccessful, as reviewed by [20]. In this study all four unchallenged but in-contact lambs developed scrapie with an incubation period of just under 2 years, very similar to natural disease in the heavily infected flock from which inoculum RBP1 was made. Precautions were taken to remove passively excreted inoculum from the pens before unchallenged lambs were mixed with dosed lambs, but a single last lamb was dosed and added to the 5 g dose group to make up numbers, and the dosing of this lamb overlapped with contact of the 4 unchallenged lambs placed in this pen. Therefore lateral transmission may have occurred via a small amount of passively shed inoculum remaining in the environment, shedding of the agent by the single last lamb, or another as yet unidentified route. It has recently been shown that Chronic Wasting Disease in mule deer in the USA is transmitted laterally not maternally, but in that study the source of contamination leading to lateral transmission was not identified and could have been placenta [27]. A similar observation has been made in natural scrapie, in which the incubation period of scrapie in sheep was similar whether born to likely infected or probably uninfected ewes, again in this case transmission via the environment contaminated at lambing time is feasible [8].

All of the control animals given brain material from a UK scrapie free flock (not the same flock as the source of the recipient lambs, but a native UK flock in which scrapie has never been reported) developed disease with a mean age at death of 1085 days. This is considerably longer than the challenged animals, and longer than for unchallenged lambs in direct contact with the challenged lambs, and indeed longer than naturally occurring scrapie in this genotype in a naturally high scrapie-incidence sheep flock [8]. The source of infection in these sheep is obscure, given the precautions taken to prevent cross-contamination between groups. It is possibly due either to undetected levels of infection in the control inoculum or spread from challenged sheep. The control inoculum was derived from two sheep, negative by immunohistochemistry on the medulla oblongata at the level of the obex. Western blotting of this inoculum has given negative results. The second possibility is that spread has occurred via trace amounts of contaminated bedding etc., despite careful precautions to avoid any contact between control and infected sheep. The source of this spread could be either residual inoculum or infection shed by the infected sheep into the environment. Whichever is the case the amount of infection transferred was extremely small, indicating that the efficiency of transmission of scrapie was far higher than previously thought. This suggests that short periods of contact between sheep, such as during transport and at markets, must be considered possible opportunities for transmission under natural conditions.

The results of this study show that experimentally induced scrapie, using a natural route of challenge in young lambs, provides a reliable and convenient model in which to study the dynamics of uptake and dissemination of prions in the natural host. Using this model we have demonstrated the existence of a latent period during which undetectable replication of the agent must be occurring, followed by local accumulation and replication that continues throughout the incubation period. Spread is sequentially as a result of drainage in efferent lymph, haematogenous spread and spread via parasympathetic and possibly sensory nerve fibres. It is suggested that afferent lymph dendritic cells provide a potential vehicle for the spread of the scrapie agent within a host. Clinical disease was associated with accumulation of disease specific prion protein in the CNS, but not vacuolar pathology.

## Methods

### Sheep

Sheep used in this study were TSE free, derived originally from New Zealand and bred in a dedicated isolation facility in the UK to ensure no contact with TSE infection. Susceptible genotypes of sheep maintained in this facility for over six years (up to the end of the current studies) have shown neither clinical signs of scrapie nor evidence of infection in routine screening of the brains of sheep culled from the flock. Lambs were produced by embryo transfer from super-ovulated susceptible genotype ewes and carried in recipient ewes of the same scrapie-free status. All lambs were of the Cheviot breed and homozygous for the VRQ allele of the sheep PrP gene (valine at codon 136, arginine at codon 154 and glutamine at codon 171). Lambs aged approximately 14 days were randomly allocated to three treatment groups; two groups were challenged orally with classical scrapie brain homogenate and one group with negative control brain homogenate. As the optimal dose was unknown two different doses, either 1 g or 5 g, of inoculum RBP1 (see below) were given undiluted to 25 and 28 lambs respectively (Table 1) by placing inoculum into the mouth directly from a syringe barrel, enabling it to be swallowed naturally. Control sheep (n = 29) were challenged in the same way using 3 g of a homogenate made from the brains of 2 clinically and histopathologically normal sheep derived from a scrapie unaffected flock in the UK.

All lambs were maintained indoors in a dedicated facility under normal sheep husbandry conditions and were weaned at 6 weeks of age. Sheep were bedded on wood shavings and pens cleaned out every other day for 28 days post challenge to reduce the possibility of passively excreted inoculum causing repeated infection. After this period sheep were bedded on straw, and their diet supplemented with cereal based concentrate prepared on-site using meat and bone meal free constituents derived from TSE-free sources. Twenty-eight days after the 14 day old lambs were dosed with positive brain homogenate, four unchallenged lambs were mixed in the pens with the lambs to test for lateral transmission. They remained with the dosed sheep until clinical end-point. Control sheep were housed in the same building as challenged sheep, but in pens separated by a 2 m gap ensuring no physical contact with challenged sheep. Care was taken to avoid cross contamination between challenged and control sheep by changing of outer clothing before entering any pens and use of dedicated feeding, cleaning and husbandry equipment for each group.

All inoculations were carried out in accordance with the UK Animal (Scientific Procedures) Act 1986, under Licence from the UK Government Home Office (Project licence no: 70/5155). Such licence is only granted following approval by the internal VLA ethical review process as mandated by the Home Office.

### Inoculum

Sheep scrapie brain pool RBP1 consists of a homogenate of the whole brains of 17 sheep euthanased in the terminal stages of scrapie in which disease was confirmed by histopathological examination of the brain stem. The sheep all originated in the institute's own flock of naturally scrapie infected sheep and comprised a mixture of VRQ and ARQ homozygotes and heterozygotes. Titration of this inoculum in RIII mice has given a titre of 10^3.96 ^mouse i.c/i.p LD_50_/g.

### Necropsies

Sheep were euthanased by intravenous injection of pentabarbitone at pre-determined time points as detailed in Table 2, or as soon as they showed definite clinical signs of scrapie. Incubation periods given are the dates to death at the onset of definite clinical signs (equivocal signs suggestive of scrapie were noted earlier than this). Tissues were collected into 10% neutral buffered formalin. Small intestinal tissues were collected within 2 minutes of death for optimal mucosal preservation. Tissues were processed and embedded in wax using routine histological procedures after 5 days fixation.

### Immunohistochemistry

5 μm sections were cut and immunolabeled with rat monoclonal antibody R145, kindly provided by Mr R Jackman, VLA-Weybridge, using a DAKO Techmate automated immunolabeller and counterstained using haematoxylin. Using this method patterns of disease specific immunlabeling are similar to published descriptions [6] and no immunolabeling is detected in normal tissue, therefore PrP detected in challenged animals is considered abnormal, termed disease specific PrP.

### Western blotting

using a modification of the Prionics-check test was performed on fresh obex samples as previously described [17].

## Authors' contributions

SJR conceived and led the project, interpreted the pathology and drafted the manuscript; GED managed the experimental work, sample collection, pathology interpretation and contributed to the manuscript; LH managed the sheep facilities, assisted with sample collection and contributed to the manuscript; RW assisted with sample collection, pathology interpretation and contributed to the manuscript; SJM performed pathology interpretation, contributed to the manuscript, and generated the Figures. All authors read and approved the final manuscript.

## References

[B1] Wilesmith JW, Wells GA, Cranwell MP, Ryan JB (1988). Bovine spongiform encephalopathy: epidemiological studies. Vet Rec.

[B2] Will RG, Ironside JW, Zeidler M, Cousens SN, Estibeiro K, Alperovitch A (1996). A new variant of Creutzfeldt-Jacob disease in the UK. Lancet.

[B3] Prusiner SB (1991). Molecular biology of prion diseases. Science.

[B4] Hunter N (1998). Scrapie. Mol Biotechnol.

[B5] Hadlow WJ, Kennedy RC, Race RE (1982). Natural infection of Suffolk sheep with scrapie virus. J Infect Dis.

[B6] van Keulen LJ, Schreuder BE, Meloen RH, Mooij-Harkes G, Vromans ME, Langeveld JP (1996). Immunohistochemical detection of prion protein in lymphoid tissues of sheep with natural scrapie. J Clin Microbiol.

[B7] Andreoletti O, Berthon P, Marc D, Sarradin P, Grosclaude J, van KL (2000). Early accumulation of PrP(Sc) in gut-associated lymphoid and nervous tissues of susceptible sheep from a Romanov flock with natural scrapie. J Gen Virol.

[B8] Ryder S, Dexter G, Bellworthy S, Tongue S (2004). Demonstration of lateral transmission of scrapie between sheep kept under natural conditions using lymphoid tissue biopsy. Res Vet Sci.

[B9] Ryder SJ, Spencer YI, Bellerby PJ, March SA (2001). Immunohistochemical detection of PrP in the medulla oblongata of sheep: the spectrum of staining in normal and scrapie-affected sheep. Vet Rec.

[B10] Heppner FL, Prinz M, Aguzzi A (2001). Pathogenesis of prion diseases: possible implications of microglial cells. Prog Brain Res.

[B11] Dickinson AG (1976). Scrapie in sheep and goats. Front Biol.

[B12] Foote WC, Clark W, Maciulis A, Call JW, Hourrigan J, Evans RC (1993). Prevention of scrapie transmission in sheep, using embryo transfer. Am J Vet Res.

[B13] Tuo W, O'Rourke KI, Zhuang D, Cheevers WP, Spraker TR, Knowles DP (2002). Pregnancy status and fetal prion genetics determine PrPSc accumulation in placentomes of scrapie-infected sheep. Proc Natl Acad Sci USA.

[B14] Heggebo R, Press CM, Gunnes G, Lie KI, Tranulis MA, Ulvund M (2000). Distribution of prion protein in the ileal Peyer's patch of scrapie-free lambs and lambs naturally and experimentally exposed to the scrapie agent. J Gen Virol.

[B15] Jeffrey M, Ryder S, Martin S, Hawkins SA, Terry L, Berthelin-Baker C (2001). Oral inoculation of sheep with the agent of bovine spongiform encephalopathy (BSE). 1. Onset and distribution of disease-specific PrP accumulation in brain and viscera. J Comp Pathol.

[B16] Bellworthy SJ, Hawkins SA, Green RB, Blamire I, Dexter G, Dexter I (2005). Tissue distribution of bovine spongiform encephalopathy infectivity in Romney sheep up to the onset of clinical disease after oral challenge. Vet Rec.

[B17] Chaplin MJ, Barlow N, Ryder S, Simmons MM, Spencer Y, Hughes R (2002). Evaluation of the effects of controlled autolysis on the immunodetection of PrP(Sc) by immunoblotting and immunohistochemistry from natural cases of scrapie and BSE. Res Vet Sci.

[B18] Wood JL, McGill IS, Done SH, Bradley R (1997). Neuropathology of scrapie: a study of the distribution patterns of brain lesions in 222 cases of natural scrapie in sheep, 1982–1991. Vet Rec.

[B19] van Keulen LJ, Schreuder BE, Meloen RH, Poelen-van den BM, Mooij-Harkes G, Vromans ME (1995). Immunohistochemical detection and localization of prion protein in brain tissue of sheep with natural scrapie. Vet Pathol.

[B20] Hoinville LJ (1996). A review of the epidemiology of scrapie in sheep. Rev Sci Tech.

[B21] Houston EF, Halliday SI, Jeffrey M, Goldmann W, Hunter N (2002). New Zealand sheep with scrapie-susceptible PrP genotypes succumb to experimental challenge with a sheep-passaged scrapie isolate (SSBP/1). J Gen Virol.

[B22] van Keulen LJ, Schreuder BE, Vromans ME, Langeveld JP, Smits MA (1999). Scrapie-associated prion protein in the gastrointestinal tract of sheep with natural scrapie. J Comp Pathol.

[B23] Yan C, Xin-Ming Q, Li-Kun G, Lin-Lin L, Fang-Ping C, Ying X (2006). Tetrandrine-induced apoptosis in rat primary hepatocytes is initiated from mitochondria: caspases and endonuclease G (Endo G) pathway. Toxicology.

[B24] Herrmann LM, Baszler TV, Knowles DP, Cheevers WP (2002). PrP(Sc) is not detected in peripheral blood leukocytes of scrapie-infected sheep: determining the limit of sensitivity by immunohistochemistry. Clin Diagn Lab Immunol.

[B25] Hunter N, Foster J, Chong A, McCutcheon S, Parnham D, Eaton S (2002). Transmission of prion diseases by blood transfusion. J Gen Virol.

[B26] Andreoletti O, Lacroux C, Chabert A, Monnereau L, Tabouret G, Lantier F (2002). PrP(Sc) accumulation in placentas of ewes exposed to natural scrapie: influence of foetal PrP genotype and effect on ewe-to-lamb transmission. J Gen Virol.

[B27] Miller MW, Williams ES (2002). Detection of PrP(CWD) in mule deer by immunohistochemistry of lymphoid tissues. Vet Rec.

